# Early Kidney Damage in a Population Exposed to Cadmium and Other Heavy Metals

**DOI:** 10.1289/ehp.11641

**Published:** 2008-09-09

**Authors:** Laura D. K. Thomas, Susan Hodgson, Mark Nieuwenhuijsen, Lars Jarup

**Affiliations:** 1 Small Area Health Statistics Unit, Imperial College London, London, United Kingdom; 2 Institute of Health and Society, Newcastle University, Newcastle, United Kingdom; 3 Centre for Research in Environmental Epidemiology, Institut Municipal d’Investigació Mèdica, Centro de Investigación Biomédica en Red de Epidemiología y Salud Pública, Barcelona, Spain

**Keywords:** cadmium, environmental exposure, heavy metals, kidney disease, nephrotoxicants, zinc smelter

## Abstract

**Background:**

Exposure to heavy metals may cause kidney damage. The population living near the Avonmouth zinc smelter has been exposed to cadmium and other heavy metals for many decades.

**Objectives:**

We aimed to assess Cd body burden and early signs of kidney damage in the Avonmouth population.

**Methods:**

We used dispersion modeling to assess exposure to Cd. We analyzed urine samples from the local population (*n* = 180) for Cd (U-Cd) to assess dose (body burden) and for three biomarkers of early kidney damage [*N*-acetyl-β-d-glucosaminidase (U-NAG), retinol-binding protein, and α-1-microglobulin]. We collected information on occupation, intake of homegrown vegetables, smoking, and medical history by questionnaire.

**Results:**

Median U-Cd concentrations were 0.22 nmol/mmol creatinine (nonsmoking 0.18/smoking 0.40) and 0.34 nmol/mmol creatinine (nonsmoking 0.31/smoking 0.46) in non-occupationally exposed men and women, respectively. There was a significant dose–response relationship between U-Cd and the prevalence of early renal damage—defined as U-NAG > 0.22 IU/mmol—with odds ratios of 2.64 [95% confidence interval (95% CI), 0.70–9.97] and 3.64 (95% CI, 0.98–13.5) for U-Cd levels of 0.3 to < 0.5 and levels ≥ 0.5 nmol/mmol creatinine, respectively (*p* for trend = 0.045).

**Conclusion:**

U-Cd concentrations were close to levels where kidney and bone effects have been found in other populations. The dose–response relationship between U-Cd levels and prevalence of U-NAG above the reference value support the need for measures to reduce environmental Cd exposure.

Over the last century, emissions of cadmium from various industries and the combustion of waste and fossil fuels have resulted in a considerable elevation of the concentrations in European soils (Järup et al. 1998). Exposure to Cd in the general population is already close to the critical level, particularly in susceptible population groups and populations living close to polluting industries.

The biological half-life of Cd is very long, in the order of decades (Järup et al. 1998). The Cd concentration in urine (U-Cd) is mainly influenced by the body burden, and U-Cd is proportional to the concentration in the kidneys, until the onset of kidney damage (Järup et al. 1998). During long-term exposure, the concentration of U-Cd increases slowly and in proportion to the amount accumulated in the body.

It is well established that exposure to high levels of Cd may cause kidney damage leading to renal failure (Järup et al. 1998). The initial sign of Cd-induced renal lesions is tubular proteinuria, usually detected as an increased excretion of low-molecular-weight proteins, such as α-1-microglobulin (A1M), retinol-binding protein (RBP), and the enzyme *N*-acetyl-β-d -glucosaminidase (NAG). There is increasing evidence that early tubular damage may develop at low cumulative Cd doses (U-Cd = 1–3 nmol/mmol creatinine) resulting from environmental Cd exposure (Buchet et al. 1990; Järup et al. 2000). Although these early changes to tubular function are not clinically significant in themselves, tubular damage may progress to glomerular damage and eventually to renal failure if Cd exposure is prolonged (Åkesson et al. 2005; Hellström et al. 2001; Järup et al. 1998).

Although the renal toxicity of Cd is well known, the dose–response relationship between Cd and kidney damage is still not well established at the low levels of exposure typically seen in environmentally exposed populations [European Commission Scientific Committee on Toxicity, Ecotoxicity, and the Environment (CSTEE) 2004; European Union 2007]. This uncertainty is also recognized by the U.S. Centers for Disease Control and Prevention (CDC) in the *Third National Report on Human Exposure to Environmental Chemicals* (CDC 2005), which stated that

[T]he urinary and blood cadmium levels at the 95th and 90th percentiles, respectively, approach these cited values associated with subclinical changes in renal function and bone mineral density. Further research is needed to address the public health consequences of such exposure in the United States.

When it closed in early 2003, the lead/zinc smelter in Avonmouth (southwest England) was the largest source of atmospheric Cd emissions in the United Kingdom and the biggest smelter of its kind in the world. In the year before closure, Cd stack emissions from the site totaled 978 kg, representing nearly 30% of U.K. point source emissions for that year according to the Environment Agencies Pollution Inventory (Environment Agency for England and Wales 2007). Zinc production had been taking place at the site for > 70 years, and during this time large amounts of other nephrotoxic metals, including lead, mercury, and arsenic, were also emitted. Because Cd is the most potent of these nephrotoxicants, and also has a very long biological half-life, we decided to focus on Cd in this study.

With close to 50,000 people living within 5 km of the smelter, there is concern that these emissions may have led to increased Cd exposure in the local population. It is thought that human exposure in this area may have occurred both directly through inhalation of contaminated air and indirectly through the ingestion of homegrown vegetables and house dust.

Soil sampling carried out in the vicinity of the smelter has shown a significant buildup of metal contamination in the soil up to 15 km from the smelter (Colgan et al. 2003). Soil Cd concentrations around smelting operations are closely related to the pattern of dispersion from the smelter (Hogervorst et al. 2007).

The aims of the present study were to assess Cd body burden (estimated by U-Cd) in a population sample living in a Cd-contaminated area and to assess the prevalence of early renal damage and any evidence of a dose–response relationship. This is the first study of environmental Cd exposure and early kidney damage in a U.K. population.

## Materials and Methods

We identified potential participants using the National Health Service database of patients registered with general practitioners (the Exeter System) in the area. We stratified these by age, sex, and estimated exposure before we took a random sample. We invited a total of 865 people to take part in the study. We asked participants to complete a short questionnaire regarding residential, occupational, and medical history, smoking, and consumption of home-grown vegetables, as well as any use of a private well. We also asked them to provide one spot morning urine sample; samples were frozen on the day of collection and kept frozen until analysis. Ethical approval for the study was granted by Bristol South and Central Ethics Committee, and all participants provided their written informed consent before data collection.

**Figure 1:**
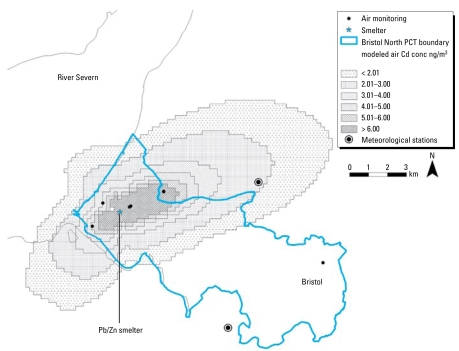
Map showing modeled air Cd concentrations (conc), the six air monitoring sites, and the site of the lead/zinc (Pb/Zn) smelter. Two of the air monitoring sites are within 100 m of each other.

We used dispersion modeling to identify the potentially exposed population and to stratify this population into high-, medium-, and low-exposure groups before recruitment. The use of air dispersion modeling as the exposure assessment method is described in Hodgson et al. (2007). We used the Atmospheric Dispersion Modelling System (ADMS)-Urban (version 2.0; Cambridge Environmental Research Consultants, Cambridge, UK) to model Cd emissions from the site between 1996 and 2002 (excluding 1997 because of the poor quality of local meteorological data for that year). During this time, up to 13 stacks were emitting Cd to air. We used emissions data collected under the Integrated Pollution Control legislation, and held by Bridgewater Public Registry, Bristol, in the model, along with stack details (height, diameter, etc.) provided by Bristol City Council. We used hourly sequential meteorological data from the Bristol Weather Centre and Filton Meteorological station; these data were provided by the British Atmospheric Data Centre (2006). Ambient air Cd concentrations in the United Kingdom are typically between 0.05 and 1.0 ng/m^3^ (Baker 2001), so we added a background concentration of 0.5 ng/m^3^ to the model. We validated the model output from the year 2000 using the annual averages from six air monitoring sites (Figure 1). The air monitoring data were provided by Bristol City Council and Stanger/National Physical Laboratory (Middlesex, England). The final model used was an average of the output for 1996–2002 (excluding 1997).

Urine samples were analyzed for Cd (U-Cd) and for three biomarkers of tubular damage [NAG (U-NAG), RBP (U-RBP), and A1M (U-A1M)]. Analysis was carried out by the Health and Safety Laboratory (HSL) in Buxton, United Kingdom. The HSL determined U-Cd using inductively coupled plasma mass spectrometry, U-NAG by fixed time incubation, U-RBP by enzyme-linked immunosorbent assay, and U-A1M by a non-competitive immunoassay. The HSL carried out all assays according to standard operating procedures; HSL participates in inter-laboratory quality control procedures. We adjusted biomarkers of both dose (U-Cd) and effect (U-NAG, U-RBP, and U-A1M) for urinary creatinine to account for differences in urine concentration. The HSL calculated reference values for U-NAG and U-RBP by the HSL using the 97.5th percentiles from a U.K. cohort of 320 working subjects with no history of exposure to nephrotoxins (Mason H, personal communication). We based the U-A1M reference level on the 95th percentile of a nonexposed, healthy Swedish population of working age (Tencer et al. 1996).

We assessed the relationship between natural log (ln) U-Cd and a set of independent variables—sex, age, ADMS category, and smoking status—in SPSS (SPSS Inc., Chicago, IL, USA) using a linear regression model. We used the median value as the representative concentration for each ADMS category.

Urinary biomarkers of both dose and effect were log-normally distributed and when plotted did not show a nonlinear relationship. We therefore used Pearson’s two-tailed correlation coefficient to test for correlation on the log-transformed data.

We calculated odds ratios for the prevalence of U-NAG above the reference level in SPSS using a logistic regression model. We chose U-Cd categories *a priori* based on the Osteoporosis—Cadmium as a Risk Factor (OSCAR) study (Järup et al. 2000). We used U-Cd < 0.3 nmol/mmol creatinine as the reference group and adjusted the analysis for sex. We used Egret software (version 3.2; Cytel Software Corp., Cambridge, MA, USA) to test for trend across the odds ratios. We assessed statistical significance at the 95% level.

## Results

Of the 865 adults invited to take part in the study, 180 (74 men, 106 women) participated (21%). The participation rate was 17% and 24% for men and women, respectively. As expected, there was an increasing response rate by age (in years: 18–29, 10%; 30–49, 15%; ≥ 50, 32%). There were no significant differences between responders and nonresponders in terms of socioeconomic status. A total of 109 participants (40 men and 69 women; 61%) were never-smokers, 36 (19 men, 17 women; 20%) had smoked in the past, and 32 (13 men, 19 women; 18%) were current smokers; smoking data were not available for three participants. Seventeen participants (15 men, 2 women) reported that they had been employed at the smelter; data on employment at the smelter were not available for three participants.

**Figure 2:**
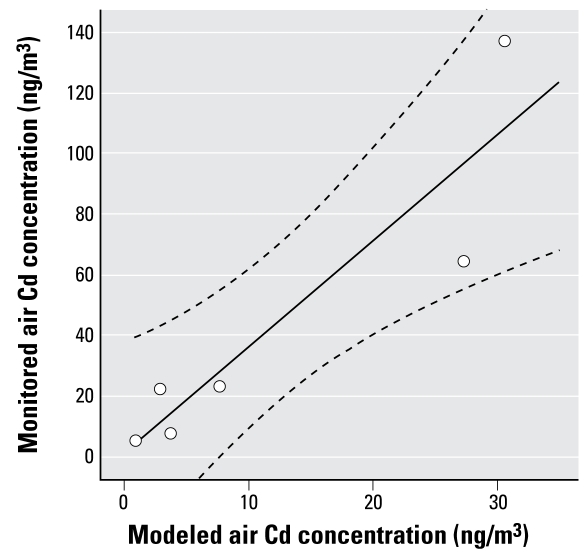
Monitored Cd levels as a function of modeled data (note different scales). Lines indicate linear regression and 95% CI (*R*^2^ = 0.84).

Figure 1 shows the modeled Cd levels and the monitoring station locations. Figure 2 shows modeled and monitored annual averages of Cd for the year 2000 for the six monitoring sites. We found that the model underestimated Cd concentrations but showed a good correlation with measured values (Figure 2). Ambient air Cd concentrations at each of the six monitoring sites exceeded the World Health Organization (WHO) *Air Quality Guidelines for Europe* (WHO 2000) of 5 ng/m^3^.

We found median U-Cd concentrations of 0.22 nmol/mmol creatinine (nonsmoking 0.18/smoking 0.40) and 0.34 nmol/mmol creatinine (nonsmoking 0.31/smoking 0.46) in nonoccupationally exposed men and women, respectively. These levels are in the same range as those found in other studies that detected early kidney damage and low bone mineral density (Table 1).

Because U-Cd was log-normally distributed, we used log-transformed values in showing U-Cd as a function of sex (female vs. male), age, ADMS-modeled Cd concentration levels, and smoking status (never, past, current smokers). We found a significant relationship between ln(U-Cd) and modeled atmospheric Cd concentrations (Table 2).

We found significant correlations between ln(U-Cd) and ln(U-NAG) for both men (Pearson’s *r* = 0.328, *p* = 0.004) and women (Pearson’s *r* = 0.399, *p* < 0.001). We also found a significant correlation between U-Cd and U-A1M in women (Pearson’s *r* = 0.220, *p* = 0.03).

**Figure 3:**
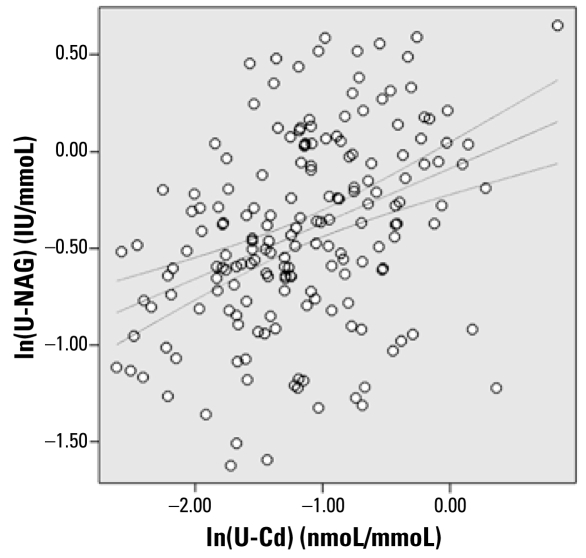
The association between ln(U-NAG) and ln(U-Cd). Lines indicate linear regression and 95% CI (*R*^2^ = 0.14).

Figure 3 shows the association between ln(U-Cd) and ln(U-NAG) for both men and women (*n* = 180; Pearson’s two-tailed *r* = 0.380, *p* ≤ 0.001). The correlation was still significant when we removed the occupationally exposed outlier (*r* = 0.357, *p* ≤ 0.001).

There was a significant dose–response relationship between U-Cd and the prevalence of U-NAG above the reference level, with odds ratios of 2.64 [95% confidence interval (95% CI), 0.70–9.97] and 3.64 (95% CI, 0.98–13.5) for U-Cd levels of 0.3 to < 0.5 and levels ≥ 0.5 nmol/mmol creatinine, respectively (*p* for trend = 0.045). The dose–response trend did not change when we excluded current and past smokers, although the total number of cases was decreased (Table 3).

## Discussion

We found median U-Cd concentrations in the Avonmouth pilot sample of 0.18 and 0.31 nmol/mmol creatinine in nonoccupationally exposed nonsmoking men and women, respectively. The corresponding data for smokers were 0.40 in men and 0.46 in women. Overall, six participants (3.3%) had U-Cd values greater than the 1 nmol/mmol creatinine level at which a 10% increased prevalence of proteinuria was found in the OSCAR study (Järup et al. 2000). Fourteen participants (7.8%) had U-Cd > 0.8 nmol/mmol creatinine, the recently revised German reference value (Wilhelm et al. 2004). Forty-five participants (25%) had U-Cd levels greater than 0.5 nmol/mmol creatinine, the lower limit of potential risk of kidney damage suggested by the CSTEE (2004).

We found significant dose–effect relationships between U-Cd and U-NAG in both men and women and a significant dose–effect relationship between U-Cd and U-A1M in women. We also found a significant dose-dependent trend in the prevalence of early renal damage, as assessed by U-NAG, and this trend persisted after exclusion of current and past smokers. The results of this study would suggest that U-NAG is the most sensitive of the biomarkers of tubular damage investigated. U-NAG is an intracellular enzyme located mainly in the lysosomes of proximal tubular epithelial cells (Fels et al. 1994). Its presence in urine therefore represents disruption of these cells. Previous studies have also identified U-NAG as a highly sensitive indicator of Cd tubular toxicity in both humans and in the animal model (Brzóska et al. 2003).

Modeled atmospheric concentrations identified populations with potential Cd exposure from the smelter site (either via direct inhalation when the smelter was operating or indirect inhalation of household dust, or via ingestion of vegetables grown on Cd-contaminated soil). There was a good correlation between ADMS-modeled levels and monitoring data, suggesting that the modeled values were valid estimates of Cd exposure from the smelter; however, we found that the model underestimated concentrations. One possible reason for this underestimation is that fugitive emissions, which are thought to have been significant, could not be included in the model because these were never quantified.

In addition to Cd, the smelter in Avonmouth also emitted arsenic, lead, and mercury, all of which are nephrotoxic. It is likely that these metals followed a pattern of dispersion very similar to that of Cd because they were emitted from the same stacks and share similar chemical properties. The similar patterns of exposure mean that it is not possible to separate the effects of Cd from those of the other nephrotoxins; our finding could therefore result from exposure to a mix of several nephrotoxic metals.

Because Cd has a long half-life in the kidney, the use of only the current residential address in the exposure estimate is likely to lead to random misclassification of exposure, such that any relationship between modeled air Cd concentrations/distance from smelter and U-Cd would be biased toward the null.

Consumption of homegrown vegetables has been identified as a probable Cd exposure pathway, most recently in a Swedish population living close to a Cd battery factory (Hellström et al. 2007). However, in the present study, we found no correlation between consumption of homegrown vegetables and U-Cd (data not shown), possibly because the questionnaire did not adequately assess the frequency of consumption, or because of the relatively small sample size. Further information on garden soil Cd concentrations and pH is needed to adequately assess the risk posed by this pathway in the Avonmouth area.

In addition to the anticipated Cd exposure from the smelter, individuals will have been exposed to Cd from other sources, in particular, from tobacco smoke, which is known to be a major source of Cd exposure. In our study, mean U-Cd levels in “ever-smokers” were approximately twice those of “never-smokers”; this is similar to findings in other studies (Järup et al. 1998).

The results identify women as a particularly susceptible group. U-Cd concentrations were significantly higher in women than in men; this difference between the sexes is well established (Vahter et al. 2007). One explanation for this difference may be that the iron deficiency commonly seen among women of childbearing age results in the up-regulation of the iron channels through which both Cd and iron are absorbed from the digestive tract (Berglund et al. 1994).

The lead/zinc smelter in Avonmouth closed in 2003, and as a result, atmospheric Cd concentrations in the area have been reduced dramatically. In the year after closure of the smelter (2004), ambient air concentrations at four of the monitoring sites (two monitoring sites were no longer in operation) were approximately 24% of those in the year before closure (2002). However, emissions from the smelter are known to have resulted in elevated soil concentrations up to 15 km from the site, and soil is therefore likely to be a continuing source of exposure in the area. Direct airborne exposure has been shown to play a minor role in the exposure of people living near a Cd-emitting plant, even when the plant was operating (Hellström et al. 2007). Indirect exposures, such as consumption of homegrown vegetables grown on contaminated soil (Hellström et al. 2007) or inhalation of contaminated household dust (Hogervorst et al. 2007), are more important pathways of exposure. It is clear that such exposures continue several decades after closure of the Cd-emitting plants (Hellström et al. 2007; Hogervorst et al. 2007).

## Conclusions

U-Cd concentrations were close to levels where kidney and bone effects have been found. Dose–response relationships between U-Cd and U-NAG prevalence support the need for measures to reduce environmental Cd exposure.

## Figures and Tables

**Table 1:** Mean U-Cd values (nmol/mmol creatinine) in European general populations (including smokers and nonsmokers but excluding occupationally exposed).

	Median U-Cd			
Population	Men	Women	Men and women	Reference
Men (*n* = 58) and women (*n* = 102), 18–86 years of age, living within 7 km of a Zn smelter.	0.18/0.40	0.31/0.46	0.25/0.44	Present study
Sweden, OSCAR study (472 women, 311 men, 16–81 years of age)	0.25/0.42	0.42/0.50		Järup et al. (2000)
Germany (4,740 adults, 18–69 years of age)			0.22/0.29^a^	Wilhelm et al. (2004)
Sweden (820 women, 53–64 years of age)		0.67^b^,^c^		Åkesson et al. (2005)
Netherlands (290 men and women)			0.34^b^	Fiolet et al. (1999)
Belgium, Cadmibel study (1,699 men and women 20–80 years of age)			0.84 μg/24 hr^b^,^d^	Buchet et al. (1990)

a Given in μg/L. Creatinine-adjusted values would depend on urine creatinine (U-Crea) concentration, which has a normal range from about 0.3 to 2–3 g/L. Thus, U-Crea = 1 g/L would give the same adjusted value, whereas higher U-Crea concentrations would give lower adjusted values. Commonly, the adjusted values are rather close to the nonadjusted values.

b Smokers and nonsmokers combined.

c Never-smokers had a U-Cd level of 0.45 μg/g.

d Geometric mean based on 24-hr sample.

**Table 2:** Multiple regression of ln(U-Cd) as a function of sex, age, ADMS category, and smoking status.

Variable	β-Value	SE	*p*-Value
Female sex	0.30	0.08	< 0.001
Age (years)	0.52	0.00	< 0.001
ADMS category (< 2, 2 to < 3, 3 to < 4, 4 to < 5, 5 to < 6, ≥6 ng/m^3^ )	0.12	0.02	0.04
Smoking status (never, past, current)	0.36	0.05	< 0.001

All variables were included in the regression model.

**Table 3:** Prevalence of U-NAG above reference level (1.25 IU/mmol creatinine) in relation to U-Cd (adjusted for sex).

U-Cd category (nmol/mmol creatinine)	Mean (nmol/mmol creatinine)	Cases/total (prevalence%)	Odds ratio (95% CI), adjusted for sex
All			
< 0.3	0.193	4/84 (4.8)	1
0.3 to < 0.5	0.381	6/51 (11.8)	2.64 (0.70–9.97)
≥ 0.5	0.777	7/45 (15.6)	3.64 (0.98–13.5) (test for trend, *p* = 0.045)
Excluding current smokers			
< 0.3	0.188	4/71 (5.6)	1
0.3 to < 0.5	0.377	5/43 (11.6)	2.32 (0.57–9.4)
≥ 0.5	0.730	6/31 (19.4)	4.25 (1.07–16.83) (test for trend, *p* = 0.033)
Excluding current and past smokers			
< 0.3	0.188	4/65 (6.2)	1
0.3 to < 0.5	0.366	2/28 (7.1)	1.55 (0.24–9.87)
≥ 0.5	0.674	4/16 (25)	8.10 (1.31–50.11) (test for trend, *p* = 0.023)
